# Application of Proximity-Labeling Techniques in Plants: A Review of Successful Cases

**DOI:** 10.3390/ijms27167227

**Published:** 2026-08-13

**Authors:** Zhiyong Yang, Shixin Yang, Qingfeng Meng

**Affiliations:** School of Biological Science and Technology, Jiangsu University, Zhenjiang 212013, China

**Keywords:** proximity labeling, plant protein–protein interaction, TurboID, PUP-IT

## Abstract

Exploring protein–protein interaction (PPI) networks during cellular processes is critical for understanding the molecular mechanisms underlying these processes. PL (proximity labeling) is an emerging technique with the potential to be a powerful protein interactomics tool. It employs proximity-labeling enzymes, coupled with mass spectrometry, to covalently label, capture, and identify interacting and neighboring proteins of the bait protein. The development of numerous novel PL enzymes and the improvement of biotin ligase-based enzymes have enabled efficient spatiotemporal mapping of PPIs, especially after the establishment of TurboID in plants. Most PL-associated reviews in plants focus on the potential applications of different enzyme-based PL. Here, we focus on PL cases effectively applied in plants and dissect each case in detail from the perspectives of PL expression design, labeling, extraction, enrichment, and quantitative proteomic identification. Moreover, we compare cases using biotin ligase-based PL (such as BioID and TurboID) and PUP-IT, highlighting the advantages and limitations of each PL system. We delineated the pipeline and optimization strategies for PL experiment design to facilitate successful execution by plant researchers.

## 1. Introduction

Proteins are essential for life and crucial to cellular functions. Proteins always function within PPI networks or protein complexes, wherein they interact and are functionally interconnected. The complexity of protein functions relies on the intricate and dynamic PPI networks [1,2]. Identifying novel PPIs for an unknown protein can reveal much more about its actual role than sequence-based predictions alone and also offers a foundation for further functional studies [3]. Plants regulate cellular functions to adapt to environmental challenges through finely tuned PPIs, thereby revealing the complex roles of core proteins and clarifying the mechanisms underlying a variety of biological processes, such as plant growth and development, adaptation to environmental challenges, and hormone signaling pathways [4]. A total of 75,000–150,000 interaction pairs are predicted from an average of 36,795 proteins in plants [5,6]. Many efforts have been made to identify the interaction pairs, such as the Arabidopsis Interactome Mapping Consortium (2011), which identified 6200 highly reliable interactions from about 2700 proteins through a large-scale PPI screening tool, yeast two-hybrid (Y2H) [7]. Thus, to accelerate advances in plant functional studies, it is urgent to identify novel PPIs in order to elucidate the mechanisms underlying key biological processes.

Protein–protein interactions can be strong or weak, permanent or transient, among which the dominant form of PPIs falls into the category of transient or weak binding events [8,9]. Moreover, transient interactions are much less conserved in their mechanisms [10,11]. Several traditional methods are commonly used for PPIs screening, including yeast two-hybrid (Y2H) and affinity purification–mass spectrometry (AP-MS). Y2H has been the predominant technique for large-scale PPI screening in plants [12,13,14]. However, Y2H is prone to producing false positives and false negatives due to artifactual protein coexpression and high expression levels, redirection of proteins to the nucleus, and non-physiological conditions [15,16,17]. Affinity purification–mass spectrometry (AP-MS) is an effective approach for PPI screening in their native physiological context, relying on the binding affinity between bait and prey proteins to pull down the prey proteins by the bait protein, in turn, by beads. Thus, the binding affinity of PPIs determines the performance of AP-MS, leading to the loss of transient and weak interactions in AP-MS data [18]. Moreover, protein–protein interaction states can vary considerably in lysate compared with those under physiological conditions within the cell. Thus, AP-MS often fails to detect interactions between membrane proteins because intact interactions cannot withstand cell lysis and the washing steps during purification [19]. Although the development of chemical crosslinking (crosslinking-based AP-MS) enables detection of transient and weak interactions by covalently crosslinking interacting proteins (along with artifactual associations) to the bait, nonspecific binding remains the major challenge [20,21]. To address these issues, new methods need to be developed to detect transient and weak PPIs, as well as other challenging PPI cases.

Proximity-labeling (PL) technology has been developed for PPI screening and has proven to be a powerful tool for identifying key interacting partners in many biological processes, especially after the establishment of TurboID [22,23,24]. Proximity labeling (PL) is a technique that uses an engineered enzyme-fused protein (a bait protein fused to a ligation enzyme with specific catalytic activity) and a cognate substrate. As the enzyme catalyzes its reaction promiscuously, it covalently attaches a substrate, such as the small molecule biotin or small peptide tags like PUP, to neighboring proteins of the bait protein. As follows, protein enrichment via affinity purification and subsequent mass spectrometric detection are performed to capture and identify neighboring proteins [22,23,25]. The PL system is implemented under native physiological conditions, maintaining interacting proteins at their inherent expression levels and specifically covalently tagging them, while avoiding nonspecific crosslinking, thereby addressing many of the limitations associated with traditional methods. The most commonly used enzymes for PL are peroxidases (APEX, HRP, APEX2) [26,27,28] and biotin ligases (TurboID, miniTurbo, BioID, BioID2, BASU) [22,23,29,30,31]. Peroxidases use the substrate biotin-phenol, while biotin ligases use biotin.

Many PL interactomics studies have been conducted over the past decade to elucidate the mechanisms underlying many core biological processes [24,32]. Although some PL interactomics studies have been carried out in plants, many challenges remain in performing PL in plants compared with mammalian cultured cells [33,34,35,36]. Therefore, optimizing the proximity-labeling system in plants is essential for its successful application. In this review, we focus on successful applications of PL in plants and dissect the experimental designs and optimization strategies used. Many other new PL techniques and applications widely used in mammalian cultured cells fall outside the scope of this review. A detailed review can be found in the references [24,32,34,36]. We dissected the cases with a focus on enzyme selection, the bait protein expression pattern (including the promoter used in the experiments, the enzyme-fusion orientation, transformation methods, and cell-type or organellar-specific expression), the control setup, and labeling time. We then summarized proteomics approaches involving labeling, including protein extraction methods, strategies for enriching tagged proteins, techniques for quantitative proteome analysis, and methods for validating candidate proteins. Lastly, we discussed the potential application of PL in plants and the challenges to PL, including DNA–protein interactions and small molecule–protein interactions, which are widely used in mammalian cultured cells but are rare in plants.

## 2. Principle and Workflow of Proximity Labeling

### 2.1. Principle

PL was developed as a complement to traditional methods [24]. During PPI screening, Y2H and AP-MS are two widely used traditional methods. Compared with Y2H, AP-MS has some advantages. First, AP-MS analyzes endogenous proteins in a native physiological context, whereas Y2H is performed in yeast. Moreover, the expression levels of interacting proteins in AP-MS are mostly at their native levels, whereas tested proteins are highly expressed in the Y2H system, leading to a high rate of false positives. AP-MS can detect multi-protein interactions, but Y2H often fails to detect them unless the bridging proteins are expressed in the yeast [37]. AP-MS also has limitations, such as its failure to detect transient and weak interactions as well as those involving plasma membrane proteins, which are often lost during cell lysis and washing steps. Crosslinking will stabilize these protein interactions at the expense of specificity, leading to a high rate of false positives [9,38]. PL can be viewed as an improved version of crosslinking-based AP-MS, in which proteins in close proximity are specifically tagged rather than randomly cross-linked. Moreover, due to the varying affinity of the bait–prey interaction, the washing buffer and conditions in AP-MS need to be optimized accordingly. PL will convert various affinities of bait–prey interactions into high-affinity interactions between tagged prey protein and affinity beads (such as the biotin–streptavidin interaction), thereby enabling the successful identification of challenging interacting proteins [39]. 

**Figure 1:**
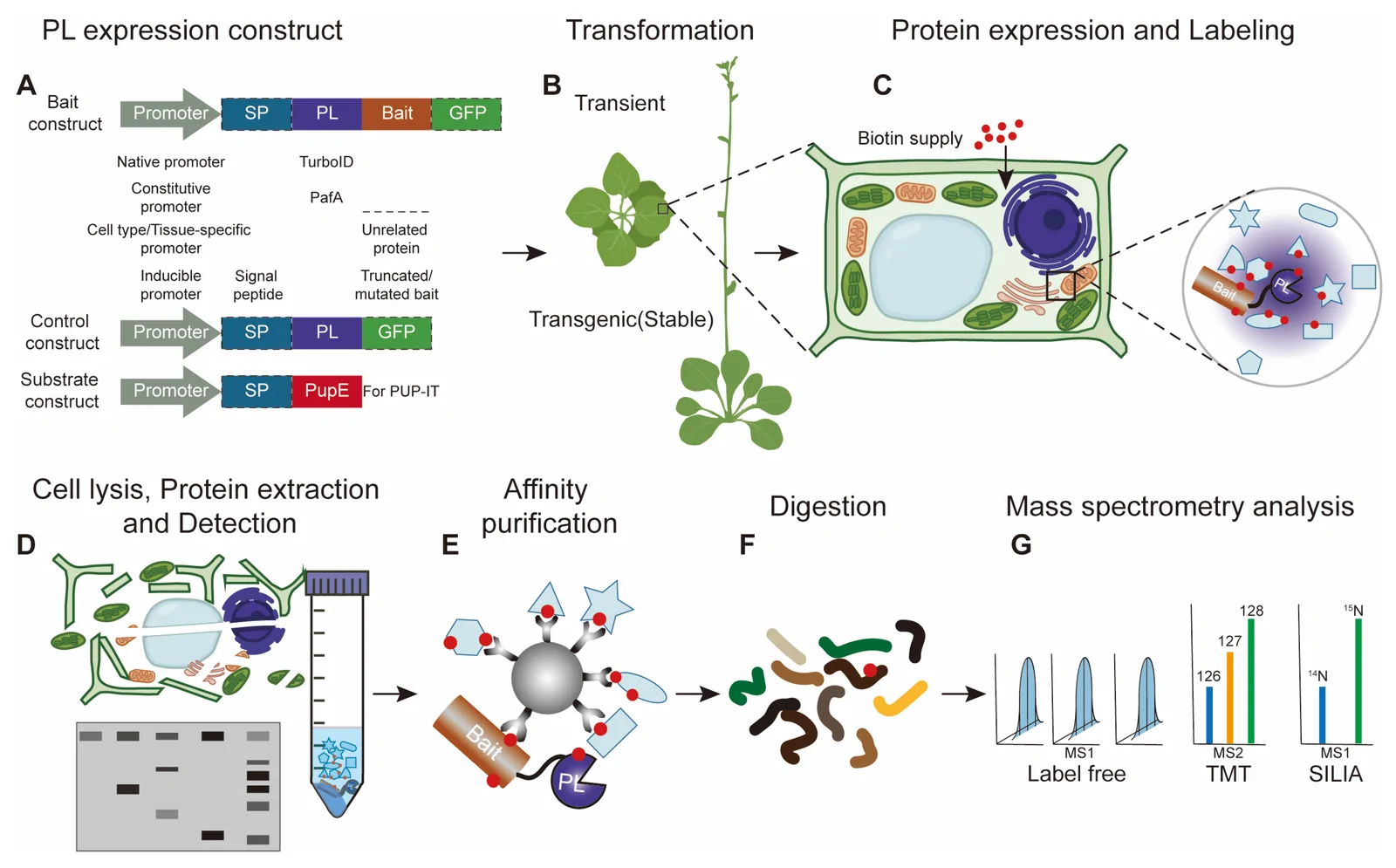
Workflow of the PL system for PPI identification in plants. The experimental pipeline consists of seven sequential steps: (**A**). PL construct design. The construct includes a promoter, localization signal peptide, PL enzyme, bait protein, and an unrelated protein (GFP). Among them, the localization signal peptide and GFP are optional in certain instances. The positions of the localization signal peptide, PL enzyme, bait protein, and GFP can be adjusted to ensure proper expression, structure, and function. Different types of promoters can be used for various purposes. In PUP-IT, the tagged PupE peptide is also expressed. (**B**). The PL constructs are introduced into plants via stable transformation (e.g., Arabidopsis) or transient transformation (e.g., tobacco leaves). (**C**). PL–bait fusions are expressed in plants, and the PL enzyme catalyzes the covalent labeling of proximal endogenous proteins (prey) within the labeling radius once the substrate (such as biotin) is present. (**D**). The endogenous proteins are released and collected during cell lysis and protein extraction. Partial samples are submitted for immunoblot detection. (**E**). Protein samples are enriched by affinity purification through magnetic beads. (**F**). Captured proteins are digested into peptides by trypsin; (**G**). Digested peptides are submitted to LC-MS/MS and analyzed by quantitative proteomic methods, such as label-free quantification, TMT labeling, and SILIA.

With the development of highly efficient PL enzymes comes a powerful tool for protein interactomics studies. A promiscuous enzyme should always be the first consideration for PL experiments, as it labels any appropriate protein without target specificity but in a proximity-dependent manner [23,31]. In PL, the engineered promiscuous enzyme is fused to the bait protein, keeping the bait and PL enzyme in close proximity. Once the substrate is present, the enzyme catalyzes the reaction by covalently attaching the substrate to neighboring proteins of the PL–bait fusion. Then, the tagged proximal proteins are enriched by affinity purification and identified by subsequent mass spectrometry (Figure 1) [24]. The enzymes engineered for PL can be broadly categorized into three main types: peroxidases, biotin ligases, and others. Among them, the most commonly used PLs are peroxidase (APEX, APEX2, HRP) and biotin ligase (TurboID, miniTurbo, BioID, BioID2, BASU), which use the diffusible substrates biotin-phenol and biotin, respectively [26,27,28,29,30]. In contrast, other enzymes (PUP-IT, NEDDylation, EXCELL, miniSOG) exhibit diversity in mechanisms, substrates, and labeling conditions [25,40,41,42]. In biotin ligase-based PL, the promiscuous biotin ligase BioID is first adapted from BirA by a mutant (R118G), turning the enzyme, which biotinylates cellular proteins with extraordinary specificity, into an engineered enzyme that promiscuously biotinylates proteins in a proximity-dependent manner [23,31]. At the beginning, the biotin ligase catalyzes the formation of the activated substrate, bio-5′-AMP, from ATP and biotin, and then the bio-5′-AMP diffuses out from the enzyme active site and covalently attaches to the ε-amino group of the target lysine in the protein, resulting in the formation of an amide bond between biotin and the lysine side chain of the target protein. The bio-5′-AMP diffuses away from the enzyme active site, with its concentration decreasing as the distance from the enzyme increases. Lower protein labeling efficiency at greater distances, due to decreased concentrations and lower stability of bio-5′-AMP, collectively defines the labeling radius (10 nm) of the biotin ligase-based PL system (Figure 1C) [29]. There were a variety of improved versions of biotin ligase developed after BioID, including BioID2, TurboID, miniTurbo, BASU. Among them, TurboID and miniTurboID were screened via yeast display-based directed evolution, with high activity at room temperature (25 °C) and largely reduced labeling time (10 min) compared with the original version BioID (37 °C, 24 h), leading to the widespread application of TurboID and miniTurboID in different organisms, especially in plants [22]. In the case of APEX, biotin-phenol and phenol derivatives are used as peroxidase substrates. Peroxidases turn the phenol substrate into an activated phenoxyl radical in the presence of H_2_O_2_, and covalently tag Tyr (mostly, and less Trp, His, and Cys) in proximal proteins and guanosine in RNA and DNA molecules. The labeling time is extremely short (1 min), and the labeling radius is about 20 nm [26]. The improved version of APEX or other peroxidase-related PL enzymes was also developed, including APEX2 and HRP [27]. Due to high cell toxicity of H_2_O_2_ and H_2_O_2_-induced stress, APEX was not successfully applied in plants. Thus, its application is outside the scope of this review.

For other PL enzymes, PUP-IT has recently been applied in plants, making it the only approach in addition to biotin ligase-based PL used in plants. PUP-IT uses the PafA (Proteasome Accessory Factor A) ligase, fused to the bait protein, to covalently attach the Pup (prokaryotic ubiquitin-like protein) peptide (64 amino acids with Gly Gly-Gln C-terminal deamination, as PupE) to a lysine residue side chain on the proximal protein [25]. The PupE peptide can be engineered by fusing an affinity tag (such as FLAG-tag or biotin) to the N-terminus for the subsequent AP-MS. During the reaction, PafA first catalyzes the phosphorylation of PupE at its C-terminal Glu to produce the reactive intermediate. The C-terminal Glu of the activated PupE-PafA complex is then conjugated to the ε-amino group of a lysine residue in the proximal protein, followed by the release of PafA from the complex. This defines the limited labeling radius of PafA, indicating that proximal protein labeling primarily depends on the linker length between PafA and the bait protein, unlike biotin ligase- and peroxidase-based PL, which utilize a diffusible activated substrate [25,43]. Moreover, all components of PUP-IT, including the PL enzyme (PafA) and substrate (PupE), are genetically encoded and expressed in the cell, unlike biotin ligase- and peroxidase-based PL, which require exogenous addition of substrate biotin or biotin-phenol.

In the case of NEDDylation, the ligase Ubiquitin-conjugating 12 (Ubc12, NEDD8 E2 ligase) is engineered and fused to the bait protein to catalyze the covalent attachment of the NEDD8 peptide (fused with a tag for AP-MS, such as a His6-biotin tag) to the proximal proteins [40]. The EXCELL was developed as a general method for detecting and recording cell–cell interactions, using an evolved Staphylococcus aureus transpeptidase, sortase A variant (mgSrtA). The mgSrtA catalyzes the covalent attachment of a small peptide (Biotin-LPXTG) to the monoglycine residue at the N-terminus of the target protein [41]. The requirement for a monoglycine residue at the N-terminus of the target protein makes it not suitable for PPI screening. As a genetically encoded photocatalyst, MiniSOG produces singlet oxygen to enable in situ covalent attachment of an exogenous nucleophilic probe to proteins, RNA, or DNA [42,44,45,46].

### 2.2. Workflow

PL techniques were initially developed and refined in mammalian cultured cells, as they are more manageable and easier to manipulate than plants. All early experimental designs were customized for mammalian cultured cells before being adapted for plant applications. Although many PL interactomics studies have been conducted in plants, their use remains limited compared with mammalian cultured cells, possibly due to the unique structural features of plants, such as a relatively small cytoplasmic volume and high levels of proteases and phosphatases (Figure 1B) [47]. The differences in PL conduction between mammalian cultured cells and plants are as follows: first, mammalian cultured cells mainly use transient transfection, whereas plants employ both transient transfection and stable transgenic lines [22,48]. Secondly, biotin supplementation strategies differ: mammalian cells are cultured in biotin-depleted medium with low-dose supplementation, whereas plants require high-concentration biotin delivered via infiltration or submergence due to the physical barriers posed by cell walls and cuticles [22,48]. Furthermore, plant PL exhibits a higher intrinsic background due to endogenous biotin and biotinylated proteins, complicating experimental optimization and data filtering compared with mammalian cultured cells [48]. Additionally, optimal growth temperatures differ between mammalian cells (37 °C) and plants (22–28 °C), which should match the optimal temperature of the specific PLs employed [22,23].

The standard workflow for plant PL can be briefly summarized as follows: Firstly, expression of PL enzyme-bait fusion in plants and PLs. Secondly, total protein extraction from plant cells and detection of labeled protein via immunoblot. Lastly, affinity purification of labeled proteins followed by mass spectrometry identification (Figure 1) [33,35,36]. For expression of PL components, constructing an expression vector according to the experimental design is the first step, among which the native promoter, constitutive promoter, cell/tissue-specific promoter, or inducible promoter was employed to express the PL enzyme-bait fusion and substrate [33]. Moreover, a signal peptide for subcellular localization and GFP for fast detection of expression are also included in the construct in some cases (Figure 1A). With the proper plasmid, the components of PL are transiently expressed in plants or protoplasts, or stably expressed in transgenic plants (Figure 1B). Then, the PL enzyme catalyzes the covalent labeling of proximal endogenous proteins (prey) within the labeling radius once the substrate (such as biotin) is present (Figure 1C). Afterward, total protein was extracted under denaturing or native conditions, followed by affinity purification using streptavidin- or antibody-coupled beads (Figure 1D,E). Biotin removal by a desalting column will be performed before affinity purification in biotin ligase-based PL. Subsequently, the final step, LC-MS/MS, including trypsin digestion, is used to identify and quantify the enriched proteins with various quantitative proteome methods, such as label-free quantification, TMT labeling, and SILIA (Figure 1F,G). During the procedures, some steps are optional, such as removing biotin using a desalting column, which was used to improve affinity purification efficiency and was applied only to biotin-involved PLs, like TurboID and APEX [36].

## 3. Dissection of the Main Application of PL in Plants

### 3.1. Choices of Enzymes

Many PL studies have been conducted to map PPI networks in plants. Over the past decade, biotin ligase-based PL methods, such as TurboID, miniTurboID, BioID, and BioID2, have remained the only PL techniques successfully applied in plants [33], and recently, the PUP-IT method has also been employed in plants, yielding high-quality results (Table 1) [43,49,50,51,52,53].

The initial version of biotin ligase-based PL BioID was employed in several plant species, including rice, Arabidopsis, and tobacco, to identify proteins that interact with transcription factors, pathogen effectors, and viral replicases, respectively [54,55,56,57,58]. Due to the optimal working temperature of 37 °C and relatively low activity, BioID exhibits slow labeling kinetics in plants, taking at least 16 h and up to 48 h for sufficient labeling, with 24 h being the most common duration. With slow labeling, it is difficult to capture PPI in real time as it occurs [23,31]. The BioID2, developed from Aquifex aeolicus BirA without the N-terminal domain of BioID, has a smaller size (27 kDa, 35 kDa for BioID) and a higher optimal temperature (50 °C) [29]. It has been employed in Arabidopsis to investigate the proximal proteins of the nuclear envelope and nuclear membrane protein [59,60].

The development of TurboID enables the rapid application of PL in mammalian cultured cells and plants, accelerating progress in various model organisms. With the highest activity of ligase and a broad working temperature, TurboID is the most commonly used PL in plants (Table 2), which also makes it the top choice for PL applications in other organisms [22]. Rapid labeling kinetics (within 10 min for mammalian cultured cells, and 15 min for plants) allow for real-time mapping of the PPI network in essential biological processes. However, high activity also comes along with an increased labeling radius (35 nm, which grows over time), which in turn compromises specificity [61]. Moreover, labeling by endogenous biotin, especially with high activity of PL enzyme, and the presence of endogenous biotinylated proteins, lead to a high background [22,48]. The major technical challenge for the application of TurboID in plants compared with mammalian cultured cells is the abundant biotin and biotinylated proteins in plants. TurboID can utilize the low levels of endogenous biotin, leading to constitutive background biotinylation even in the absence of exogenous biotin supplementation [48]. With half the activity of TurboID and a smaller size (28 kDa, 35 kDa for TurboID), mini TurboID was developed and exhibits lower background than TurboID. TurboID and mini TurboID have been successfully applied to many plant species, including Arabidopsis, tobacco, tomato, and liverwort [48,62,63,64]. The proper expression, structure, and function of the PL–bait fusion protein are also a challenge for proteins with large sizes or structure-sensitive. The smaller enzyme mini TurboID is friendly for structure-sensitive proteins. Moreover, biotin ligases are generally ineffective in oxidative or acidic compartments, such as peroxisomes and vacuoles [34].

In 2018, PUP-IT was initially developed to screen interacting proteins of membrane proteins in mammalian cultured cells [25]. Recently, PUP-IT has been widely used in plants, such as Arabidopsis, tobacco, and soybean [43,50,51,52]. The first application of PUP-IT in plants was performed in Arabidopsis mesophyll protoplasts for identifying substrates of TOR kinase, in which the fusion protein of PafA and the C-terminal kinase domain of TOR (TOR-C–PafA–HA) was co-expressed with Flag-tagged Pup(E), as well as one of the six candidate substrates (MYC-tagged PRC2 components: CLF, SWN, EMF2, VRN2, FIE, and MSI1), and only FIE was specifically pupylated. FIE was identified as interacting with TOR kinase not by standard PL workflows such as AP-MS, but via co-immunoprecipitation and Western blotting assays, demonstrating that PUP-IT serves as a powerful alternative tool for screening and verifying protein–protein interactions (PPIs) [49]. Subsequently, four applications of PUP-IT were carried out on Arabidopsis, tobacco, and soybean using a standard PL method with quantitative proteomics, further confirming its high efficiency in PPIs screening, especially in studies of plant development (FERONIA, TOR, CELLULOSE SYNTHASE complex (CSC)) and responses to stress (GmAPC/C) [43,50,51,52].

Unlike biotin-based PL, PUP-IT employs the PupE peptide as the substrate, instead of biotin, thereby avoiding the high background labeling by endogenous biotin and biotinylated proteins in plants [43]. Moreover, the labeling radius of PUP-IT largely depends on the linker length between PafA and the bait protein, approximately 60–80 Å for a 15–20-amino-acid linker [25]. Additionally, using the non-diffusive substrate, PUP-IT labeling of interactors occurs only in a confined region with enhanced specificity [25,43,51]. Several modified versions of PUP-IT were also created for specific applications, including PUP-IT2 for structurally sensitive proteins [65], SPIDER for in vitro screening of interacting proteins that bind biomolecules such as proteins, DNA, RNA, and small molecules [39], and POST-IT for identifying target proteins of bioactive molecules [66]. These versions have not been used in plants but could be considered as potential options for application. A comparison of BioID, TurboID, and PUP-IT is shown in Table 1.

For most PL applications in plants, TurboID is the top choice, with its highest activity and a broad working temperature. Moreover, when capturing dynamic interactome snapshots and transient PPIs, TurboID is preferred due to its fast-labeling kinetics [22]. When the elevated background noise caused by TurboID impedes the identification of genuine interactors, mini TurboID is preferred, considering the compromise between background interference and enzymatic activity. Furthermore, for the bait protein that exhibits sensitivity to fusion size, mini TurboID is also favored because of its smaller size relative to TurboID [22,24]. BioID and BioID2 are not favored owing to their extended incubation period. However, for experiments that suffer from high background caused by endogenous biotinylated proteins and exogenous biotin, PUP-IT is employed to capture the interacting proteins with compromised activity and temporal control. Moreover, for tissues or cells where exogenous biotin cannot penetrate well, PUP-IT is the optimal option [25].

**Table 2:** PL–bait expression pattern in plants.

PL Ligase	Plants	Expression Pattern	Promoter-PL-Bait Plasmid	Control Set	Labeling Time and Biotin Concentration	References
BioID	Rice protoplasts	Transiently transformed	pUbi::BirA*-OsFD2	pUbi::BirA* pUbi::WTBirA pUbi::BirAG-FD1	24 h with 50 µM biotin	[54]
BioID	*Transgenic Arabidopsis*	Stably transformed and DEX-induced expression	pBD::HopF2-BirA*-FLAG-HA	pBD::BirA*-FLAG-HA	24 h with 30 μM DEX and 2 mM biotin	[55]
BioID	*Nicotiana benthamiana* leaves	Transiently transformed	pT70::AvrPto-BirA-MP	pT70::BirA	24 h with 75 µM biotin	[56]
BioID	TMV-infected *Nicotiana benthamiana* leaves	Transiently transformed	pXY::BirA*-126k-EGFP pXY::126k-BirA*-EGFP	pXY::BirA*-EGFP	48 h with 0.5 mM biotin	[57]
BioID	TMV-infected *Nicotiana benthamiana* leaves	Transiently transformed	pXY01::mCherry-HA-BirA*-ATG8	PXY01-Cherry-HA-BirA*	72 h with 2.5 mM biotin	[58]
TurboID	*Nicotiana benthamiana* leaves	Transiently transformed	p35S::gN-TurboID-HA p35S::N_TIR-TurboID-HA	p35S::Citrine-TurboID-HA	15 min with 200 µM biotin	[62]
TurboID	*Transgenic Arabidopsis*	Stably transformed	pFAMA::FAMA-TbID-Venus	pFAMA::TbID-YFP_NLS_, WT (non-transformed control)	0.5 h and 3 h with 50 µM biotin	[48]
BioID2	*Transgenic Arabidopsis*	Stably transformed	p35S::HA-BioID2-SUN1 p35S::HA-BioID2-NEAP1 p35S::HA-BioID2-WIP1 p35S::HA-BioID2-SINE1	p35S::HA-BioID2-Nup93a p35S::YFP-BioID2	24 h with 50 μM biotin	[59]
BioID2	*Transgenic Arabidopsis*	Stably transformed	p35S::HA-BioID2-SINE1 p35S:: PUX5-BioID2	p35S::HA-BioID2-WIT1 p35S::YFP-BioID2	16 h with 50 µM biotin	[60]
TurboID	*Arabidopsis* cell suspension cultures	Stably transformed	p35S::TPLATE-BioID p35S::TPLATE-BioID2 p35S::TPLATE-(GGGGS)_13_-BioID/BioID2 p35S::TPLATE-(GGGGS)_13_-TurboID	p35S::GFP-BioID p35S::GFP-(GGGGS)_13_-TurboID	24 h with 50 µM biotin	[67]
TurboID	Transgenic *Arabidopsis*	Stably transformed	pXPO4::XPO4-3HA-TurboID	Treated with water pXPO4::XPO5-3HA-TurboID pXPO4::XPO7-3HA-TurboID	6 h with 50 µM biotin	[68]
TurboID	*Nicotiana benthamiana* leaves	Transiently transformed	p35S:: NtPhyt-Turbo-His_6_	p35S::SP-TurboID-His_6_	5 h with 200 µM biotin	[69]
Mini TurboID	*Marchantia polymorpha* transgenic lines	Stably transformed	pMpSYP13B:: miniTurboID-Myc-MpSYP13B pMpSYP12A:: miniTurbo-Myc-MpSYP12A	Wild-type Tak-1	24 h with 700 µM biotin.	[70,71]
PUP-IT	*Arabidopsis* mesophyll protoplasts	Transiently transformed	pUBQ10::TOR-C-PafA-HA p2×35S::3×Flag-pup (E)	pUBQ10::PafA-HA p2×35S::3×Flag-pup (E)	12 h incubation	[49,53]
TurboID	Transgenic *Arabidopsis* leaves	Stably transformed	p35S::MPK4-TurboID-2×HA	vector control	3 h with 50 μM biotin	[72]
TurboID	*Arabidopsis* PSB-D cell culture	Transiently transformed	p35S::TurboID-SnRK1α1-3×HA p35S::SnRK1α1-TurboID-3×HA p35S::TurboID-SnRK1βγ-3×HA p35S::SnRK1β1-TurboID-3×HA p35S::TurboID-SnRK1β1-3×HA p35S::TurboID-SnRK1β2-3×HA	wild-type transgenic cultures expressing an unrelated TurboID fusion protein	6 h or 24 h with 50 μM biotin	[73]
TurboID	Transgenic *Arabidopsis*	Stably transformed	p35S::BIN2-YFP-TurboID	p35S::YFP-YFP-TurboID	3 h with 50 µM biotin	[74]
TurboID	Transgenic *Arabidopsis*	Stably transformed	pASY1::ASY1-eYFP-TbID pASY3::eYFP-TbID-ASY3	pASY1::ASY1-eYFP pASY3::eYFP-ASY3 pUBQ10::_NLS_eYFP-TbID	6 h with 0.5 mM biotin using the stem-cut method	[75]
TurboID	Transgenic *Arabidopsis*	Stably transformed	p35S::DCP1-TurboID-6xHis-3xFLAG	p35S::GFP-TurboID-6xHis-3xFLAG	24 h with 50 µM biotin	[76]
TurboID	*Nicotiana benthamiana* leaves	Transiently transformed	p35S::P23-Citrine-TurbolD	p35S::2C1-Citrine-TurbolD	8 h with 200 µM biotin	[77]
TurboID	*Chlamydomonas reinhardtii*	Stably transformed	pPSAD::RBCS2-TurboID-HA pPSAD::EPYC1-TurboID-HA	Wild type pPSAD::RPE1-TurboID-HA pPSAD::PRK1-TurboID-HA	4 h with 2.5 mM biotin	[63]
TurboID	*Chlamydomonas reinhardtii*	Stably transformed	pAR::VIPP1-TurboID pAR::VIPP2-TurboID	pAR::cpCDJ1-mCherry-TurboID	4 h with 1 mM biotin	[64]
TurboID Mini TurboID	Transgenic *Arabidopsis*	Stably transformed	p35S::3HA-EGFP-mTurboID-OMP25 p35S::3HA-mTurboID-EGFP-OMP25 p35S::ABCD1-TurboID-EGFP-3HA p35S::ABCD1-EGFP-TurboID-3HA p35S::TOC64-III-TurboID-EGFP-3HA p35S::TOC64-III-EGFP-TurboID-3HA	p35S::EGFP-TurboID Wild type	1 h with 100 µM biotin	[78]
TurboID-RNA IP	Transgenic *Arabidopsis*	Stably transformed	p35S::DCP1-TurboID-6xHis-3xFLAG	p35S::sfGFP-TurboID-6xHis-3xFLAG	24 h with 50 µM biotin, and 15 min with 1% (*v*/*v*) formaldehyde	[79]
TurboID	*Petunia* protoplasts	Transiently transformed	p35S::C4H-TurboID-EGFP, p35S::C4H-TurboID,	p35S::ER-TurboID-EGFP	0 h or 3 h with 50 µM biotin	[80]
TurboID	Transgenic *Arabidopsis*	Stably transformed and estradiol-induced expression	pXVE::MKK1-TurboID-Hisx8 pXVE::MKK2-TurboID-Hisx8	pXVE::TurboID-Hisx8	With 150 μM biotin and with 5 μM estradiol	[81]
TurboID split-TurboID	*Nicotiana benthamiana*	Transiently transformed	p35S:SGT1-TurboID-Myc p35S:SGT1-HA-TurboID^N^ p35S:HSP90-Myc-TurboID^C^	p35S:Citrine-TurboID-Myc	8 h with 200 mM biotin	[82]
TurboID	*Arabidopsis* PSB-D cell culture	Transiently transformed	p35S::TurboID-SnRK1α1-3×HA p35S::SnRK1α1-TurboID-3×HA p35S::SnRK1β1-TurboID-3×HA p35S::TurboID-SnRK1β1-3×HA p35S::TurboID-SnRK1β2-3×HA p35S::SnRK1β3-TurboID-3×HA p35S::TurboID-SnRK1βγ-3×HA p35S::TurboID-Raptor1B-3×HA p35S::LST8-1-TurboID-3×HA	WT p35S::GFP-TurboID-3×HA	15 min or 1 h with 50 µM biotin	[83]
PUP-IT	*Arabidopsis* protoplasts, seedlings, and flowers	Transiently and stably transformed. Estradiol-induced expression	p35SPPDK::FER-PafA-HA p35SPPDK::6xhis-3xflag-pupE pFER::FER-PafA-HA pXVE::6xhis-3xflag-pupE	pUBQ10::PafA-HA pHBT::6xhis-3xflag-pupE transgenic line without induction of FLAG-pupE	For protoplasts, 12 h after transfection For seedlings, 48 h after estradiol induction For flowers, 72 h after estradiol induction.	[43]
PUP-IT	Transgenic *Arabidopsis*	Stably transformed and estradiol-induced expression	pUBQ10::LST8-1-PafA pUBQ10::RAPTOR1-PafA pUBQ10::ScFKBP-PafA p35S::FLAG-PUP(E) pXVE::FLAG-PUP(E)	pUBQ10::eGFP-PafA	16 h and 36h after 10 μM *β*-estradiol induction	[50]
PUP-IT	Transgenic *Arabidopsis*	Stably transformed	pUBQ10::PafA-CC1 pCC1::PafA-CC1	pUBQ10::PafA-LTI6B	Constitutive expressed	[51]
PUP-IT	Transgenic soybean	Stably transformed and DEX-induced expression	pGmUBI::GR-LhG4 pOp6::PafA-3×HA-GmILPA1/APC8 pOp6::GmAPC10-PafA-3×HA pOp6::GmCDC20-PafA-3×HA pOp6::3×FLAG-pup	pOp6::PafA-3×HA	24 h with 20 μM DEX	[52]
CSPL (TurboID-dCas9)	Transgenic *Arabidopsis* Cabbage Rice	Stably transformed	pAtUbi10::NLS-dCas9-TurboID pU6-26::sgRNA(pPIF4) pU6-29::sgRNA(pPIF4)	non-targeting sgRNA biotin-untreated samples	3 h with 100 μM biotin	[84]

### 3.2. PL Expression Pattern

Two strategies are used in plants to express PL components: transient transformation, which delivers the expression cassette into plant cells for short-term expression without genomic integration; and stable transformation, which integrates the cassette into the host genome for long-term, heritable expression [67].

Transient transformation is the most favored method in the initial stage of plant PL application due to its rapid and efficient expression (Table 2) [54,56,57,58,62]. Tobacco is the most commonly used plant for transient expression of the PL system [48,56,57,58,62,67]. Additionally, Arabidopsis protoplasts and suspension cells are also utilized for such transient expression studies. Not all plants are appropriate for transient expression due to their low transformation efficiency [85]. Additionally, transient expression typically does not fully reflect the plant’s inherent physiological processes and cannot accurately replicate the phenotype of the bait protein in PL. This phenotype match confirms the functional relevance of bait–PL fusion with the endogenous bait protein, which is the prerequisite for using PL [24]. However, transient expression was still used in proof-of-concept studies for PL system optimization and rapid screening of interacting proteins due to its speed and efficiency [48,62,67]. Furthermore, transiently expressed proteins are typically produced at high levels and are not affected by position effects or gene silencing, which are the main challenges in stable protein expression [86]. Although transient expression does not always recapitulate native expression patterns, there are some strategies for mimicking the native expression patterns. Firstly, employing the native promoters with the native cis-regulatory elements is a useful strategy [43]. Secondly, agroinfiltration conditions, including the agrobacterium dosage, can be adjusted to reduce the overexpression. Lastly, functional phenotype validation can be used to assess the quality of transient expression [48,67]. For conditions that cannot function effectively in tobacco leaves, we strongly recommend that researchers utilize transgenic lines for more accurate results.

A stable transformation will deliver numerous benefits. Firstly, it is conducted under native physiological conditions and will exhibit a phenotype similar to that of the bait gene. Secondly, once PL–bait fusion is expressed and validated through phenotypic analysis, it will produce sufficient material at any required developmental stage for future experiments and will be accessible for various treatments [87]. For stable transformation, the main challenge is achieving high-level expression of the PL–bait fusion, as low-level expression can result from multiple factors, including gene silencing, an important mechanism that attenuates protein expression. Moreover, it is labor-intensive and time-consuming, requiring selection and clonal isolation [88].

In a stably transformed plant, the bait protein is fused to a PL enzyme at either its N- or C-terminus, and in some cases a GFP protein or a GFP-derived protein is also fused for fast detection of protein expression (Figure 1A) [48,57,58,74,75,77,78,80]. It is highly recommended to use the native promoter for driving PL–bait expression to accurately mimic the plant’s natural expression context [43,68,70,75]. However, continuous expression driven by a constitutive promoter—such as the CaMV 35S or Ubiquitin10 promoter—is commonly used in successful PL applications (Table 2), although its high expression level may lead to phenotypes different from the native context, or to degradation of the PL–bait proteins [89,90]. The primary reason is that, in some cases, the low expression level of PL–bait under the native promoter is insufficient either to induce phenotypic changes or to achieve high-level labeling of proximal proteins for subsequent MS identification. For instance, Zheng et al. reported that native promoter-driven PL–bait fusion partially complemented the mutated phenotype, whereas Ubiquitin10 promoter-driven protein fusion complemented the phenotype more effectively [51]. A functional complementation analysis of the bait mutant is an efficient way to validate PL–bait function compared with the endogenous bait before its further use. Still, many cases using native promoters in PL studies yielded promising data for further functional research [70,71,75]. For example, the guard cell-specific FAMA promoter was used as the native FAMA promoter for the guard cell-specific proteome study, yielding low expression relative to a constitutive promoter but sufficient labeling for subsequent MS identification [48]. A tissue/cell-type-specific promoter is a viable option for proteome studies targeting specific tissues or cell types. The inducible promoter was also employed to control PL–bait fusion expression, thereby managing the labeling process, such as HopF2-BioID, MKK1/2-TurboID [55,81]. Moreover, in PUP-IT, the inducible promoter is commonly used to regulate labeling via triggering expression of either the substrate alone or both the substrate and the PL–bait fusion, simulating the effect of exogenous biotin supply in TurboID [43,50,52].

### 3.3. Subcellular Proteome

Eukaryotes have evolved specialized compartments and organelles, both membrane-bound and membraneless, to carry out biological processes separately and efficiently within their exclusive space [91,92]. For the subcellular proteome, the PL enzymes were fused to localization peptides targeting different organelles/subcellular structures of the plant cell, or were fused directly to resident proteins from different subcellular locations. Subsequently, all proteins that share the same localization are in close proximity and, in turn, are labeled, thereby yielding the subcellular proteome [60,78,80]. Compared with traditional methods such as organelle-specific and whole-cell biochemical fractionation using centrifugation or detergents, the PL-based subcellular proteome avoids contamination and inaccuracies by providing precise labeling [93]. Moreover, PL methods have a unique advantage in studying subcellular proteomes associated with membraneless structures, such as LLPS-assembled biomolecular condensates, which have been reported to regulate many biological processes and are among the most popular research topics [94,95]. Two studies related to processing bodies in plants demonstrate successful identification of novel interactors using TurboID [76,79]. A diffusible substrate such as biotin can penetrate most organelles effectively to label proteins within compartments [64]. A non-diffusive substrate, like PupE, needs to be expressed in the specific compartment as a bait protein using the appropriate localization signal instead of penetrating, which has not yet been used in subcellular proteome studies but would be better specifically localized than biotin [36].

### 3.4. Control Setup and Labeling Time

Since PL enzymes label proteins in a proximity-dependent manner, there is a chance of tagging a protein that occasionally occupies a position within the labeling radius, or of labeling a protein that contacts the reactive substrate that has diffused out of the labeling radius, leading to incidental, nonspecific background labeling [35,96]. Moreover, endogenous biotinylated proteins, proteins commonly labeled by most baits, and nonspecific-binding proteins are important sources of background labeling [97]. To minimize these background labelings, it is crucial to include appropriate controls. Moreover, quantitative proteomics with multiple biological replicates is highly recommended and should be performed alongside a proper control to obtain high-quality PL data [24,98]. A proper control is selected accordingly (Figure 1A), which may include the PL enzyme alone [54,55], the PL enzyme fused to a localization peptide, or the PL enzyme fused to an unrelated protein (such as GFP) or a mutated version of the bait [48,57,58,62]. Additionally, in certain instances, the untransformed wild-type plant, or transgenic lines not treated with either biotin or the inducer chemical, served as negative controls (Table 2) [60,68,73,83]. Among them, the PL enzyme fused to an unrelated protein (such as GFP) is the commonly used negative control.

Labeling yield determines the quality of AP-MS identification and is influenced by various factors, including PL enzyme activity, abundance, and labeling duration, since insufficient labeling yield leads to poor enrichment and loss of interactors in MS identification [22]. To enhance labeling yield, extending the labeling duration is an effective approach: simply harvest the sample at appropriate time points after biotin treatment to allow labeling to accumulate [31]. With higher labeling yield, there is also an increase in background labeling. The labeling time ranges from several minutes to dozens of hours to obtain improved labeling yield and reduced background [24]. It is all about finding the right balance between labeling yield and background during experimentation optimization. Sometimes, higher labeling yield is favored over lower background noise.

For BioID and BioID2 applications in plants, the labeling time varies from 16 to 72 h, which is a relatively longer time compared with other PL methods (Table 2) [58,60]. The low activity of the BioID enzyme results in inefficient labeling, which requires longer labeling times and attenuates the capture of dynamic PPIs. Moreover, BioID exhibiting low labeling efficiency necessitates a higher expression of the PL–bait fusion to achieve effective labeling. In plants, the labeling duration for TurboID and mini TurboID varies from 15 min to 24 h [62,67]. Due to the diffusive nature of activated biotin, background labeling of nonspecific proteins is inevitable, especially for enzymes with high activity, like TurboID. A short duration (less than one hour) of biotin treatment is highly recommended to capture the PPI in real-time, but in many cases, the labeling time exceeds 3 h (Table 2), which may compromise labeling specificity [48,62,67,99]. However, this yields sufficient biotinylated proteins for subsequent MS analysis, especially for low-abundance PL–bait fusion proteins. Furthermore, the native endogenous biotin in plants is vulnerable to the highly active biotin ligase, leading to increased leaky labeling of proteins in the absence of biotin treatment.

For PUP-IT used in plants, the labeling time is mainly controlled by an inducible promoter that uses small molecules, such as estradiol and dexamethasone (Table 2), which activate either the expression of the substrate alone or both the substrate and PL–bait fusion by regulating transcription [43,50,52]. The labeling time, controlled by the inducible promoter, ranges from 12 to 48 h. Moreover, in the case of plants using a constitutive promoter, the labeling process lasts throughout the life of the plant, from the expression of the substrate and PL–bait fusion until sample harvest, under conditions similar to leaky TurboID labeling [51] and how they can be interpreted from the perspective of previous studies and of the working hypotheses. The findings and their implications should be discussed in the broadest context possible. Future research directions may also be highlighted.

## 4. Proteome Methods for PL

### 4.1. Protein Extraction

Cell lysis buffer typically consists of a buffer (such as Tris-HCl buffer) to maintain proper pH, a detergent (like Triton X-100 or SDS) to break down cells by solubilizing membrane lipids and proteins, and a protease inhibitor to prevent protein degradation. For biotin ligase-based PL systems, the enrichment of biotinylated proteins can be conducted under fully denaturing conditions without affecting the extremely high affinity (Kd = 10^−14^ M) between streptavidin and biotin [100]. Therefore, in many cases, using BioID or TurboID, 8 M urea or 6 M guanidine-HCl was added to the buffer, which significantly improves the efficiency of protein extraction compared to other buffers, especially for proteins derived from poorly soluble compartments and those of low abundance, such as membrane proteins [23]. PUP-IT techniques are incompatible with 8 M urea or 6 M guanidine-HCl when using anti-FLAG bead enrichment in plants [43,51]. Therefore, the biotin ligase-based PL (BioID and TurboID) outperforms PUP-IT in the protein extraction step.

### 4.2. Enrichment via Affinity Purification

During biotin treatment in biotin ligase-based PL, a high concentration (over 50 μM) of biotin was supplied to the plant cells (Table 2). After labeling, a considerable amount of free biotin remains in the plant cells. During protein extraction, free biotin will be released from plant cells. This results in protein lysis with high concentrations of biotin, which will competitively bind to streptavidin beads and significantly compromise the affinity purification of biotinylated proteins labeled in the PL process [33]. Thus, removal of free biotin prior to streptavidin bead-based protein enrichment is essential to improve the enrichment of biotinylated proteins. For PUP-IT, there is no need to remove the substrate before affinity purification due to its limited endogenous concentration.

Following labeling, proteins will be enriched through affinity purification and identified using mass spectrometry. Effective enrichment plays a crucial role in ensuring high-quality PL performance. For most of the successful PL cases in plants, enrichment was performed at the protein level (Table 3), using streptavidin beads or anti-FLAG beads to capture the biotinylated proteins and FLAG-tag labeled proteins, respectively [22,43,67]. For biotin ligase-based PL, the extremely high affinity (Kd = 10^−14^ M) of streptavidin–biotin binding tolerates highly harsh conditions during cell lysis, binding, and washing procedures, such as fully denaturing conditions utilizing 8 M urea or 6 M guanidine-HCl [101]. AP-MS significantly benefits from the use of denaturing conditions, guaranteeing highly purified samples [38]. Under denaturing conditions, the efficiency of protein extraction is significantly improved. Moreover, during the washing step, the denaturing buffer was used to eliminate nonspecific binding, significantly enhancing the specificity of the affinity purification process. However, the high affinity between biotin and streptavidin prevents efficient elution of biotinylated proteins, and the elution step cannot be performed using the usual competitive binding method. Streptavidin enrichment combined with on-bead trypsin digestion is typically the most effective elution approach (Table 3) [23,29]. Since the streptavidin–biotin complex is largely resistant to digestion by Lys-C and trypsin, biotinylated peptides remain attached to the streptavidin beads after digestion, leading to loss of most biotinylated peptides during MS identification [48,67,102]. In addition, in-gel digestion was also used to process the biotinylated protein samples eluted by boiling in Laemmli sample buffer (SDS loading buffer) [59,60,68,72].

For PUP-IT, elution was performed by competitive binding (Biotin for strep-tag), by denaturing buffer (2 M or 8 M urea for FLAG-tag), or by boiling in Laemmli sample buffer (for both strep-tag and FLAG-tag), followed by in-solution digestion or in-gel digestion [43,50,51,52]. Furthermore, due to its compatibility with trypsin digestion, the Pup-tagged peptide is detectable by MS, providing direct evidence of labeling and revealing topological information and surface-exposed residues of the candidate PPIs.

### 4.3. Quantitative Proteome

The quantification methods of MS in the PL system determine the identification and statistical significance of enriched proteins. In PL cases within plants, three methods were used: label-free quantification (LFQ), multiplexed tandem mass tag (TMT) isobaric labeling, and ^15^N metabolic labeling. The most commonly used method in plant PL applications is label-free quantification (LFQ) (Table 3), which offers the advantages of low cost, ease of operation, and no limit on the number of samples [55,67]. However, the downside lies in reduced sensitivity and increased replicate variance. LFQ relies on mass spectral peak intensities, or spectral counting, of peptide signals detected at the MS1 level [103]. Unlike in labeled methods like TMT, protein samples in LFQ are measured individually, leading to increased variance in replicates due to deviations across many processes, such as sample preparation and instrumentation. Thus, more replicates are required to obtain statistically significant results, and the cutoff of statistical significance will be lower than that of the labeled methods [22,98]. Additionally, in LFQs, extra fractionation steps for complex samples are not allowed.

Multiplexed tandem mass tag (TMT) isobaric labeling is increasingly used in plant PL studies for its high accuracy [54,62,63,77,82]. All samples labeled using TMT reagent with different mass reporters were collectively measured in a single run of mass spectrometry, reducing replicate variance. Quantification at the MS2 level provides improved accuracy and sensitivity over other MS1 methods. Moreover, TMT allows extensive fractionation, increasing the coverage of protein identification. Meanwhile, with the commercial labeling kit for up to 35 samples, the cost of TMT is comparatively higher [104].

Metabolic stable isotope labeling, such as ^15^N metabolic labeling, uses a strategy of incorporating stable isotope salts into plant proteins through metabolism in vivo, named stable isotope labeling in Arabidopsis (SILIA), which is quite different from SILAC in mammalian cultured cells. There were several cases using ^15^N metabolic labeling in PL plant applications [74,81]. For protein quantification in ^15^N metabolic labeling, ^14^N and ^15^N-containing salts are used to label proteins in the control and PL–bait sample, respectively, leading to a mass difference in the MS1 peaks of the same peptide in the control and PL–bait sample, of which the abundance ratios are used for relative protein quantification. After in vivo labeling, plant samples containing PL–bait fusion and control are harvested, mixed, and subjected to protein extraction, thereby significantly reducing replicate variance. Moreover, extensive fractionation of complex samples will improve protein identification coverage. However, for sufficient labeling, at least 16 days of plant growth on medium containing ^15^N nitrogen prior to biotin treatment is required [74].

### 4.4. Validation of Candidate Interactors

Proximity labeling captures proteins within the diffusion range of reactive substrate, encompassing both physical interactors and spatially adjacent proteins that do not interact with bait proteins [22,23,24]. The identification of known interactors and the discovery of novel candidate interactors both serve the purpose of the PL method. Before conducting further studies, the candidate interactor needs to be validated. There are two approaches for this validation. Initially, examining the phenotypes of knock-out or overexpression lines of the candidate interactors will aid in validating their function and provide important functional relevance to the bait protein [51,52,62]. Secondly, validation of PPIs between the bait protein and candidate interactors was conducted using bimolecular fluorescence complementation (BiFC), yeast two-hybrid assays, Co-IP, and PL methods, confirming the physical PPIs in vivo and in vitro [49,62,74]. Both validations above are prerequisites for further functional studies of candidate interactors.

## 5. Comparison of TurboID and PUP-IT

With the ongoing development of various PL methods, only three have been successfully implemented in plants. BioID was the first PL method developed and the first method used in plants [23,54]. With the advancement of the high-activity BioID variant, TurboID, the application of PL in mammalian cultured cells and plants was accelerated [22,62]. TurboID is the most widely used PL in plants, recognized for its high activity (Table 2). Recently, a novel PL method, PUP-IT, has been developed and implemented in plants, suitable for plants due to its genetically encoded substrate, which can be designed for biotin-free applications [25,49]. Compared to TurboID, PUP-IT has its advantages and limitations. Here, we summarized the differences between the two main PL techniques applied in plants. On the upside, firstly, PUP-IT is free of an exogenous supply of biotin to plants and avoids the background labeling from endogenous biotin and biotinylated proteins from plant cells [51]. Secondly, PUP-IT can directly identify the labeled peptides in the MS data. Moreover, PUP-IT has been reported to exhibit higher specificity than TurboID [36]. All PUP-IT components are genetically encoded, enabling subcellular proteome studies by directing the PL enzyme and substrate to specific locations precisely via the localization signal peptide, avoiding the targeted penetration of biotin into specific compartments for TurboID [36]. On the downside, the main challenge is that PUP-IT exhibits lower labeling activity than TurboID, requiring more time to reach adequate labeling levels for MS and preventing real-time mapping of the interaction network [49]. Secondly, PUP-IT utilized in plants demonstrates reduced efficiency in protein extraction and affinity purification in comparison to TurboID [22,43,62], due to its biotin-free nature, which circumvents streptavidin–biotin enrichment. Thirdly, the larger size (54 kDa) of PafA in PUP-IT can complicate the expression, structure, and function of the PL–bait fusion protein [65]. To position PUP-IT as a competitive PPI-screening tool comparable to TurboID, several improvements are necessary. Firstly, a high-affinity tag can be chosen for affinity purification to increase efficiency and specificity. Secondly, low activity levels can be enhanced through directed evolution of pafA, as with TurboID and APEX, although achieving the optimal version might be challenging [22,27]. Moreover, to address the size problem, a small version of PafA is required, albeit with the PUP-IT2 [65]. PUP-IT provides another option for PL screening of interacting proteins in plants, aiming to address problems that were previously difficult to access.

## 6. Conclusions and Future Prospects

PL technique is a powerful tool for mapping dynamic interaction networks with high spatial and temporal resolution in their native conditions, particularly for transient or weak PPIs. With numerous applications of PL methods in plants, an increasing number of novel PPIs have been identified, facilitating exploration of many mechanisms that were previously difficult to access with traditional methods, such as plasma membrane-localized proteins, apoplast-localized proteins [69], stomatal cell-specific proteome [48], chloroplast/mitochondrial membrane-localized proteins [63,78], and components of processing bodies [76]. Every method has its advantages and limitations. PL captures proximal proteins within a defined diffusion radius, including direct physical interactors, bystander proteins that are near the bait but do not interact with it, and freely diffusing cytoplasmic proteins [22,23,24]. Thus, PL may capture indirect interactors and has a high chance of false positives, with the main source from the latter two above. However, PL can generate highly specific results when employing quantitative proteomics and incorporating appropriate controls for statistical analysis [24]. Moreover, the expanding effective labeling radius coming with elevated ligase activity, increased biotin concentration and longer incubation time, may reduce labeling specificity, therefore impairing the use of TurboID [48,67]. Overexpressing a bait–PL fusion may yield sufficient labeling for key physiologically relevant findings compared with native expression, but the nonspecific labeling of proteins increases while the expression levels of the PL enzymes rise [67]. PL methods might not be appropriate for plants under all conditions and could fail to identify known interactors in some cases for various reasons. Applying PL methods to plants poses many challenges, despite numerous successful applications. Lysines on the prey protein surface are the prerequisite for PL, and PL enzymes can have preferences for labeling certain endogenous protein substrates. Moreover, steric hindrance, linker length, and cellular environments (for instance, pH, ATP concentration, and temperature) may affect the labeling process, necessitating optimization to maximize the utility of PL in plant research.

There is significant potential to explore PL applications in plants beyond PPIs, such as split-TurboID [82], TurboID-RIP for protein-RNA interaction [79], CSPL (TurboID-dCas9) for protein-DNA interaction [84], could offer new insights into biological processes after adaptation for plant research [82]. While TurboID has been successfully deployed in Arabidopsis, tobacco, tomato, maize, and citrus, extending the approach to the non-model plant species remains challenging [48,67]. The efficacy of TurboID and other biotin ligases in other plant species and organelles remains to be conclusively established [34]. Possible barriers are as follows: first, low transformation efficiency may impede delivery of the PL–bait fusion construct into plant cells. Secondly, the efficiency of biotin penetration into tissues and biotin-related background, such as endogenous biotin and biotinylated proteins, may vary among plant species. Moreover, secondary metabolites, divergent cellular pH, and redox conditions may interfere with the labeling processes. Lastly, the expression, structure, and function of PL–bait fusion in non-model plant species may vary accordingly, necessitating practice optimization [67]. It is a wonderful opportunity to expand our toolkit and deepen our understanding of plant biology.

DNA–protein interactions play a crucial role in regulating gene transcription, constituting the molecular basis of many biological processes, particularly in plants. Various PL methods have been employed to identify proteins that interact with the target DNA in mammalian cultured cells [105,106,107,108], but only one study has been conducted in plants [84]. With the adaptation, PL can be widely used in plants to screen transcription factors that regulate the expression of target genes under specific conditions, such as biotic or abiotic stress or developmental stages. In the PL case involving protein–DNA interactions in plants, the TurboID enzyme was fused to dCas9 (dead Cas9 from the CRISPR system) to map DNA–protein interactions, with a single-guide RNA (sgRNA) targeting dCas9 to a specific genomic locus of the bait DNA. Next, the proximal proteins of the bait DNA were biotinylated, captured by streptavidin beads, and then identified by MS. Using this method, Zhang et al. identified known and novel upstream-interacting proteins on the PIF4 promoter in Arabidopsis, cabbage, and rice [84]. In plants, this method can target dCas9 to any specific genomic locus and will be used to reveal novel regulatory mechanisms underlying many biological processes, such as stress response, development, and growth, thereby accelerating the improvement of critical agricultural traits. Furthermore, this method can also be employed to investigate the mechanism of emerging genome editing tools applied in plants. For instance, the mechanism of soybean cytidine base editor (ChyCBE), whose activity is significantly enhanced by fusion with a GmRad51 DNA-binding domain, can be explored by targeting TurboID to the ChyCBE target locus [109].

Moreover, the use of PL to study small molecule–protein interactions in plants has only recently started to be explored. Based on biotin ligase, PROCID, BioTAC, and DrugID were developed for small molecule–protein interaction studies in mammalian cultured cells [110,111,112]. The main difference between PL application in small molecule–protein interactions and in PPI is the creation strategy of bait-PL fusion. In PROCID, BioTAC, and DrugID, researchers use the strategy where they bind Halo tag-PL fusion or SNAP tag-PL fusion to the bait molecule-Halo/SNAP ligand, forming a bait molecule-PL complex, among which the PL enzymes TurboID, mini TurboID, and BASU were employed, respectively [110,111,112]. As small molecule–protein interactions occur at short distances with the bait molecule, a smaller radius of PL is preferred [32]. While based on PUP-IT, which has a smaller labeling radius, SPIDER and POST-IT are also developed for studying small molecule–protein interactions in mammalian cultured cells. POST-IT uses the strategy of binding Halo tag-PL fusion to the bait molecule-Halo ligand to create a bait molecule-PL complex [66]. In the case of SPIDER, the purified PafA catalyzes the covalent attachment of the streptavidin-PupE fusion protein to the proximal protein of the biotinylated bait molecules. These bait molecules occupy a proximal position on the purified streptavidin-PupE through the streptavidin–biotin interaction [39]. These methods can be easily adapted for use in plants with minor adjustments, although no methods have been applied in plants yet. In the case of methods using Halo/SNAP-tag, such as PROCID, BioTAC, DrugID and POST-IT, the main challenge is delivering the Halo/SNAP ligand-labeled small molecule into plant cells through the plant cuticle, cell wall, and plasma membrane, compared with mammalian cultured cells. A key limitation of the in vitro SPIDER method is the high risk of losing critical interactions, as maintaining the intact intracellular environment is vital for them. These methods demonstrate great potential for investigating the signaling mechanisms of plant metabolites, such as plant hormones.

Combining TurboID with a conditionally stable GFP-binding protein (destabilized GBP, a 14 kDa nanobody) will create a GFP-dependent PL system, which has been successfully implemented in zebrafish, termed BLITZ (Biotin Labeling In Tagged Zebrafish). In the BLITZ system, the dGBP-PL becomes stabilized exclusively in the presence of bait-GFP/GFP, which binds to the dGBP-PL fusion protein, resulting in the formation of the bait–PL complex for conducting the labeling process; otherwise, the dGBP-PL fusion protein will be degraded by the ubiquitin–proteasome system when it is not bound to GFP fusion [113]. The method will be highly advantageous for plant research, as numerous transgenic plants expressing GFP fusions have been generated for functional studies. Achieving PL can be straightforward by crossing dGBP-PL transgenic lines with existing bait-GFP transgenic lines, thereby preserving the transgenic lines with proper protein expression, structure, and function for second use, especially for those that are difficult to generate. Additionally, this can be accomplished by transiently expressing the bait-GFP fusion protein in dGBP-PL transgenic lines, or by expressing the dGBP-PL fusion protein in bait-GFP transgenic lines for plants compatible with transient expression, such as tobacco. This transient approach enables control of the labeling process through GFP fusion expression, similar to how exogenous biotin supply does in TurboID; thus, it is suitable for PUP-IT to manipulate the labeling process. Adapting this system to plants for mapping the interactome will enable the reuse of GFP fusion transgenic plants, as well as save the time and effort required to generate PL–bait transgenic plants.

## Figures and Tables

**Table 1:** Characteristics of PL enzymes effectively utilized in plants.

PL Type	Enzymes	Size (kDa)	Labeling Time	Labeling Radius	Substrate
Biotin ligase	BioID	35	16–48 h	10 nm	Biotin (diffusive)
	BioID2	27	16–24 h	10 nm	
	TurboID	35	15 min–24 h	10–35 nm	
	miniTurboID	28	15 min–24 h	10–35 nm	
PUP-IT	PafA	54	6–24 h	Direct, 60–80 Å for 20-amino-acid linker	Tagged-PupE (non-diffusive)

**Table 3:** Proteome strategies for PL in plants.

PL Ligases	Plants	Quantification Strategies	Enrichment Strategies	Bait Protein and Localization	Key Findings	References
BioID	Rice protoplasts	6-plex TMT isotope labeling	Streptavidinbead enrichment and On-bead digestion	Transcription factor OsFD2, which is localized in-cell nucleus.	12 putative OsFD2 neighboring proteins were identified, including putative BRI1-KD-interacting protein 128, GTP-binding proteins, histone and ribosomal proteins.	[54]
BioID	Transgenic *Arabidopsis*	Label-free quantification	Streptavidinbead enrichment and On-bead digestion	Type III effector HopF2, which is targeted predominantly in the plant plasma membrane.	19 putative HopF2-interacting proteins were identified, including known targets (such as RIN4, REM1.3, PCAP1, PHOT2) and novel candidates (such as SYP122, PHOT1, PMI1, At5g16730, At1g10140, At5g55860).	[55]
BioID	*Nicotiana benthamiana* leaves	Label-free quantification	Streptavidinbead enrichment and On-bead digestion	The effector AvrPto, which is targeted to the plasma membrane of plant cells.	Five AvrPto proximal plant proteins were identified, including RIN4, APK1, TOM1, At4g14200, APK2 (At3g22750).	[56]
BioID	TMV-infected *Nicotiana benthamiana* leaves	Label-free quantification	Streptavidinbead enrichment and On-bead digestion	TMV replicases 126 kDa, which was colocalized in the cytoplasm of N. benthamiana leaf.	18 replicase 126 kDa proximal proteins were identified, including NbSGT1, PR-10, A1YUL9, ATP-dependent RNA helicase, glycine cleavage system P protein, fructose-bisphosphate aldolase, and photosystem II protein D1/D2.	[57]
BioID	TMV infected *Nicotiana benthamiana* leaves	Label-free quantification	Streptavidinbead enrichment and On-bead digestion	The core autophagy protein ATG8, which is localized on autophagosomes membrane.	A total of 67 proteins were identified, including 16 known interacting proteins and 14 novel candidate proteins related to plant defense and autophagy, such as HYPK, ATG3, calmodulin, subtilisin-like protease, Acyl-CoA-binding domain-containing protein 3.	[58]
TurboID	*Nicotiana benthamiana* leaves	TMT labeling	Streptavidinbead enrichment and On-bead digestion	The N TIR-NLR receptor, which was observed in the nucleus and in the cytoplasm.	371 (gN) and 256 (N-TIR) proteins were identified in the presence of p50, including NbUBR7, NbPPR, NbR1B-14, NbRGA1, NbRPP13L, NbR1A-3, NbRGA3, NRC2a.	[62]
TurboID	Transgenic *Arabidopsis*	Label-free quantification	Streptavidinbead enrichment and On-bead digestion	Cell-type-specific transcription factor FAMA, which was localized in the nuclei of stomatal cells.	47 ‘high confidence’ FAMA interactor candidates were identified, including TFs and transcriptional co-regulators. Among the TFs, ICE1, three other bHLH TFs (AIB/JAM1, JAM3, and BIM1), and the non-canonical bHLH-type TF BZR1 were identified. Among the transcriptional co-regulators, MED16, HAC1, TPR3, TPR4 and SEU/LUG/LUH co-repressor complex were identified.	[48]
BioID2	Transgenic *Arabidopsis*	Label-free quantification	Streptavidinbead enrichment and in-gel digestion	Plant nuclear envelope (NE) transmembrane protein SUN1, NEAP1, WIP1, SINE1	A total of 72 proteins with high confidence were identified by the four baits, including CRWNs, KAKU4, WIT1, WIT2, RanGAP1, RanGAP2 and 15 additional PNET candidates (such as, TMEM18, kinase-like protein encoded by AT3G46280).	[59]
BioID2	Transgenic *Arabidopsis*	Label-free quantification	Streptavidinbead enrichment and in-gel digestion	Inner nuclear membrane (INM) proteins	A total of 15 SUN1-specific preys were identified, including CRWN1, CRWN2, CRWN3, CRWN4, KAKU4, PUX4, PUX5 UFD1C, UFD1B, PNET7. 132 candidates were identified by PUX5, including PUX3, PUX4, CRWN4, CDC48A, CDC48B, UFD1B, UFD1C, and NPL4A.	[60]
TurboID	Arabidopsis cell suspension cultures	Label-free quantification	Streptavidinbead enrichment and On-bead digestion	Plasma membrane-associated octameric complex	Significant interactors, including other seven TPLATE complex members, two Clathrin Heavy Chains (CHCs) and several Dynamin-Related Proteins (DRPs), SCAMP5 PICALM3, PICALM4A, TOL6 and TOL9, were identified. TurboID identified most of the TPC subunits more robustly compared with BioID2 and BioID2, but with the lower signal:noise ratio.	[67]
TurboID	Transgenic *Arabidopsis*	Label-free quantification	Streptavidinbead enrichment and In-gel digestion	Nuclear transport receptor XPO4	A total of 244 candidates were identified, including seven FG Nups (Nup214, Nup98a, Nup82, Nup54, Nup50a, Nup50b, and Nup50c) and four TPL and TPR proteins (TPL, TPR1, TPR2, and TPR4).	[68]
TurboID	*Nicotiana benthamiana* leaves	Label free quantification	Streptavidinbead enrichment and In-gel digestion	Plant subtilase phytaspase, which is secreted into the apoplast, and transport from the apoplast back into plant cells.	Several candidate phytaspase interactors were identified in this study, including Endoplasmin, BiP, Rubisco (large subunit), Calreticulin-3, and Saposin B type domain-containing protein.	[69]
Mini TurboID	*Marchantia polymorpha* transgenic lines	Label-free quantification	Streptavidinbead enrichment and On-bead digestion	Plasma membrane-localized SNARE proteins MpSYP12A and MpSYP13B	A total of 637 and 602 proteins, including MpCSR1, were identified as potential interactors of MpSYP12A and MpSYP13B, respectively. ‘Vesicle-mediated transport’ was the most significantly enriched GO term for both interactome data.	[70,71]
PUP-IT	*Arabidopsis* mesophyll protoplasts	Label-free quantification	FLAG-bead enrichment and In-gel digestion	C-terminal kinase domain of TOR (target of rapamycin) kinase	FIE was specifically pupylated by TOR-C–PafA–HA.	[49,53]
TurboID	Transgenic *Arabidopsis*	Label-free quantification	Streptavidinbead enrichment and In-gel digestion	Mitogen-activated protein kinase MPK4, which is localized in cytoplasm.	93 candidate MPK4 interacting proteins were identified in 1-1 or 1-4 TurboID transgenic samples, including MKK1.	[72]
TurboID	*Arabidopsis* PSB-D cell culture	Label-free quantification	Streptavidinbead enrichment and On-bead digestion	SNF1-related kinase 1 SnRK1, which is localized in cytoplasm, nucleu and ER.	SnRK1 interactome of 152 PPIs among 120 proteins were identified.	[73]
TurboID	Transgenic *Arabidopsis*	SILIA-MS	Streptavidinbead enrichment and On-bead digestion	GLYCOGEN SYNTHASE KINASE 3 family protein BIN2, which is localized in the nucleus and in the cytoplasm.	482 BIN2-proximal proteins were enriched, including BSL1, BSL2, BSL3, BSK3, PP2A, TOPLESS (TPL), TPR1, TPR2, TPR4), BZR1, BZR2/BES1, cellulose synthase and the MAPK kinase YODA (YDA).	[74]
TurboID	Transgenic *Arabidopsis*	Label-free quantification	Streptavidinbead enrichment and On-bead digestion	The two meiotic chromosome axis proteins ASY1 and ASY3, which are localized in the nucleus.	A total of 39 candidate proteins were identified, including ASY4, REC8, PDS5b, ZYP1a, ZYP1b, PCH2, PRD3.	[75]
TurboID	Transgenic *Arabidopsis*	Label-free quantification	FLAG-bead enrichment, Streptavidin-bead enrichment and On-bead digestion	The conserved component of processing bodies, DCP1, which is localized in the plasma membrane	The PB core components were enriched in the PDL but not in AP, including RBP47 and RH52. SCAR–WAVE components like PIR121/SRA1, NAP1, and GRL/NAP125 were also highly enriched in PDL datasets, especially upon heat stress.	[76]
TurboID	*Nicotiana benthamiana* leaves	TMT labeling	Streptavidinbead enrichment and On-bead digestion	The viral replication protein p23, which is localized in the ER membrane.	185 p23-proximal proteins were enriched in the presence or absence of BBSV infection, including the reticulon family proteins RTNLB2, RTNLB4 and heat shock protein 70 (Hsp70).	[77]
TurboID	*Chlamydomonas reinhardtii*	TMT labeling	Streptavidinbead enrichment and On-bead digestion	Rubisco small subunit 2 RBCS2, Rubisco linker protein EPYC1, which are both localized in the chloroplast.	84 unique proteins were identified in “pyrenoid proxiome”, which contains 14 out of 19 known pyrenoid components, and 30 proteins were enriched in “high-confidence pyrenoid proxiome”, which contains most known pyrenoid proteins (11/14) found in the former candidate pools.	[63]
TurboID	*Chlamydomonas reinhardtii*	Label-free quantification	Streptavidinbead enrichment and In-gel digestion	VESICLE-INDUCING PROTEIN IN PLASTIDS 1 and 2 (VIPP1 and VIPP2), which are both localized in the chloroplast.	39 VIPP1/2 interacting proteins were significantly enriched, including HSP70B, HSP70C, CDJ2, and CLPB3, CHLP1 and LPA3.	[64]
TurboID Mini TurboID	Transgenic *Arabidopsis*	Label-free quantification	Streptavidinbead enrichment and In-gel digestion	outer mitochondrial membrane-anchored protein OMP25, peroxisomal membrane protein ABCD1, chloroplast membrane protein TOC64-III	607, 542, and 616 proteins were identified in mitochondrion, peroxisome, and chloroplast proximity proteome, respectively.	[78]
Turbo-RNA IP	Transgenic *Arabidopsis*	Label-free quantification and RNA-seq	FLAG-bead enrichment, Streptavidin-bead enrichment and RNA-seq	The conserved component of processing bodies DCP1, which is localized in plasma membrane	1670 and 2723 RNAs were enriched in DCP1 samples in NS and HS conditions, respectively, which involved in-cell wall development and regeneration, plant hormonal signaling, secondary metabolism/defense, and RNA metabolism.	[79]
TurboID	*Petunia* protoplasts	Label-free quantification	Streptavidinbead enrichment and On-bead digestion	Endoplasmic reticulum (ER) membrane anchored protein C4H	185 proteins and 69 proteins were significantly enriched in samples before biotin incubation (t = 0) and in the biotin treatment (t = 3 h), respectively, including two C4H isoforms (C4Hb, C4Hc), several phenylpropanoid pathway related enzymes (DFR, RT, A3′MT and A3′5′MT, AN9, CAD, CCoAOMT, C3′H)	[80]
TurboID	Transgenic *Arabidopsis*	iBAQ quantitation and SILIA-based quantitation	Streptavidinbead enrichment and in-gel digestion	mitogen-activatedproteinkinasekinase1 (MKK1) and 2 (MKK2), which are both localized in the cytoplasm	12 and 72 proteins were significantly enriched in TurboID-MKK1 and TurboID-MKK2 samples, respectively, including WIRK1 (RAF36), DYRKP-2A, SNX2B, ACIP1, BOB1.	[81]
TurboID split-TurboID	*Nicotiana benthamiana*	TMT-labeling	Streptavidinbead enrichment and On-bead digestion	SGT1 as a positive regulator in NLR-mediated plant defenses, which is localized in cytoplasm and nucleus	In the absence or presence of p50, 298 and 196 proteins were significantly enriched in the SGT1-TurboID samples, while 141 and 169 proteins were significantly enriched in SGT1-TurboID^N^ plus HSP90-TurboID^C^ samples, including RAR1, NSL1, MIP1, SRFR1, acyl3a, MMT1, EHD1 and SRK2B.	[82]
TurboID	*Arabidopsis* PSB-D cell culture	Label-free quantification	Streptavidinbead enrichment and On-bead digestion	SNF1-related kinase 1 SnRK1, and TOR (target of rapamycin) kinase, which are both localized in cytoplasm, nucleu and ER.	229 interactions among 159 proteins were identified in the PL-MS experiments, including BSK4, RAB18, NIA1, NLP8, IRE1A, FLZs, SKINs, and class II TPS-like proteins.	[83]
PUP-IT	*Arabidopsis* protoplasts, seedlings, and flowers	Label-free quantitation	Anti-FLAG beads enrichment and in-gel digestion	FERONIA (FER), a plasma membrane-localized receptor kinase	581, 115, and 736 specific FER-interacting proteins were identified in protoplasts, seedlings, and flowers, respectively, including GRP7, PUB9, AHA2, GEF4, BAK1, PUB9, HGL1, MYC2, ANJ, HERK1, ROPGEF2, TOR, CAR10.	[43]
PUP-IT	Transgenic *Arabidopsis*	Label-free quantitation	Anti-FLAG beads enrichment and in-solution digestion	TOR complex subunits LST8-1 and RAPTOR1, ScFKBP(FK506binding protein), which are all cytosolic protein	Between 65 and 499 proteins were identified as putative TORC interactors, including 38 proteins previously reported to be TORC interactors, such as TOR, THY1 and 2, CCT1 and 2, and eIF2B subunit delta.	[50]
PUP-IT	Transgenic *Arabidopsis*	Label-free quantitation	Anti-FLAG beads enrichment and in-solution digestion	CC1 is a transmembrane protein with its N terminal facing the cytosol and C terminal in the apoplast	254 proteins were significantly enriched, including CESAs (1, 3 and 6) and CSI (1 and 3), SHOU4, SRF1, DET3, BEN1, BIG2, BIG5, AtMIN7, AtMIN10 and HLB1.	[51]
PUP-IT	Transgenic soybean	Label-free quantitation	Anti-FLAG beads enrichment and in-solution digestion	Three key subunits of the soybean APC/C, which are localized to the nucleus and cytoplasm	87, 97, and 120 enriched proteins were identified as interactors in the GmILPA1/APC8, GmAPC10, and GmCDC20 samples, including GmPEX11C, APC4, PCNA, SGT1, Rab11A, CROWDED NUCLEI 2, AMMECR1 domain-containing protein, and MPK6.	[52]
CSPL (TurboID-dCas9)	Transgenic *Arabidopsis* Cabbage Rice	Label-free quantitation	Streptavidin bead enrichment and in-gel digestion	sgRNA targeting promoter region of PIF4	A total of 99 nucleus localized proteins were enriched, including ten TFs, such as TCP, FHY3, GTL2, LSH3, and EBS, ELF7, HD2B.	[84]

## Data Availability

No new data were created or analyzed in this study, Data sharing is not applicable to this article.

## Abbreviations

The following abbreviations are used in this manuscript:

PL	Proximity Labeling
PPI	Protein–Protein Interaction
AP	Affinity Purification
